# Atypical sensors for direct and rapid neuronal detection of bacterial pathogens

**DOI:** 10.1186/s13041-016-0202-x

**Published:** 2016-03-09

**Authors:** Ji Yeon Lim, Seung-In Choi, Geunyeol Choi, Sun Wook Hwang

**Affiliations:** Department of Biomedical Sciences, Korea University College of Medicine, Seoul, 136-705 Korea; Department of Physiology, Korea University College of Medicine, Seoul, 136-705 Korea

**Keywords:** Pathogen, Bacteria, Sensory neuron, Pattern recognition receptors, TRPA1, ADAM10, FPR, Pain, Inflammation

## Abstract

Bacterial infection can threaten the normal biological functions of a host, often leading to a disease. Hosts have developed complex immune systems to cope with the danger. Preceding the elimination of pathogens, selective recognition of the non-self invaders is necessary. At the forefront of the body’s defenses are the innate immune cells, which are equipped with particular sensor molecules that can detect common exterior patterns of invading pathogens and their secreting toxins as well as with phagocytic machinery. Inflammatory mediators and cytokines released from these innate immune cells and infected tissues can boost the inflammatory cascade and further recruit adaptive immune cells to maximize the elimination and resolution. The nervous system also seems to interact with this process, mostly known to be affected by the inflammatory mediators through the binding of neuronal receptors, consequently activating neural circuits that tune the local and systemic inflammatory states. Recent research has suggested new contact points: direct interactions of sensory neurons with pathogens. Latest findings demonstrated that the sensory neurons not only share pattern recognition mechanisms with innate immune cells, but also utilize endogenous and exogenous electrogenic components for bacterial pathogen detection, by which the electrical firing prompts faster information flow than what could be achieved when the immune system is solely involved. As a result, rapid pain generation and active accommodation of the immune status occur. Here we introduced the sensory neuron-specific detector molecules for directly responding to bacterial pathogens and their signaling mechanisms. We also discussed extended issues that need to be explored in the future.

## Background

The immune system is primarily responsible for the body’s defense against invasion by bacterial pathogens. This defense mechanism usually proceeds in the following chronological order: the detection of pathogens by innate immune cells (e.g. monocytes/macrophages, neutrophils, dendritic cells, mast cell, natural killer cells); the phagocytosis of those cells and the promotion of protective changes in infected tissues, adaptive immune cells, vascular systems and nerves by intercellular communications; massive inflammation for combating pathogens; the elimination of the pathogens and tissue resolution [56]. Diverse cytokines, inflammatory mediators, degraded fragments from pathogens and dead host cells are utilized for intercellular communication. During this process, sensory neurons (somatosensory neurons when the infection occurs around the skin and autonomic sensory neurons when the infection is near the viscera) are believed to join the body’s protective mechanism. Locally, the terminals of the sensory neurons generate peptidergic transmitters such as calcitonin gene-related peptide (CGRP), Substance P and galanin which may potentiate immune cell function and enhance vascular permeability ([15]; Mcmahon et al. 2015). Furthermore, in the vagus circuit, the reflex loop is activated to tune the spleen’s function [4].

In the early stages, the activation of the immune system and of neurons by innate immune cells or infected tissues involve similar sets of messenger molecules including interleukin-1β (IL-1β), tumor necrosis factor-α (TNF-α), prostaglandins (PGs) and nerve growth factor (NGF) [see review: [15]). At neurosensory aspects, these molecules often facilitate pain perception because pain-mediating C-fiber neurons possess the receptors and downstream intracellular signaling components and accordingly excitatorily respond through them. For example, IL-1β enhances the voltage sensitivity of voltage-gated Na^+^ channel [7], TNF-α activates the sensory neuronal p38 mitogen-activated protein kinase (MAPK) pathway and facilitates neuronal excitability [55, 65], postagandins directly activate transient receptor potential (TRP) ion channels or sensitize them by G-protein coupled-protein kinase A and C phosphorylations (Moriyama et al., 2005; [3, 43, 60]), and NGF up-regulates the expression and translocation of excitatory ion channels via p38 MAPK and phosphoinositide 3-kinase (PI3K) pathways [33, 58]. Therefore, once innate immune mechanisms are initiated, they prompt somatosensory pain perception, which may help protect against further damage by driving the host to avoid aggravating the infected areas.

Again from a sensory perspective, the advantage of activating sensory neurons is the ability to generate instant responses, especially for avoidance for potential and practical dangers. The remarkably dense mesh-like structure of peripheral sensory nerve terminals that cover the tegument and most internal organs at micrometer scales and the speed of the informational transmission at millisecond scales may confer the abilities. Thus, it is not a stretch to suggest that the body could take advantage of this neuronal system for bacterial surveillance, even at the time of invasion, which would be an earlier stage than can be achieved by the elevation of inflammatory mediators and cytokines. To examine this hypothesis, one needs to determine whether the sensory system directly senses pathogens themselves or the molecules that the pathogens secrete as the innate immune cells typically do. If true, in accordance, one can see that unpleasant sensation like pain due to the nerve excitation may occur before inflammation matures.

### Evidence of direct detection of bacteria by neurons

#### When putting bacteria or their components on the nerves

Pain is one of the most important cardinal signs of inflammation produced by injury or infection. Surprisingly, information on whether bacterial pathogens cause pain via the direct stimulation of sensory neurons remains limited. The local injection of complete Freund’s adjuvant (CFA), which contains heat-killed mycobacteria, has been a useful tool to create poly-arthritic or mono-arthritic animal models during several decades. These CFA-injected models are also popular for observing chronic inflammatory pain. Despite its long history and popularity, profiles of the acute aspects of CFA-induced pain or of the in vitro sensory neuronal responses to CFA exposure have been long time out of scope. The observation periods for inflammation or pain are typically several days to months after cutaneous inoculation. It should first be considered whether the strains used in CFA (Mycobacterium tuberculosis or butyricum) are the best choices for research on pathogens that are most likely to affect patients’ pain. Nonetheless, some of the descriptions of acute CFA-evoked pain may need to be carefully revisited; for example, spontaneous paw lifts of rats, a behavioral pain parameter and their nociceptor firing 1 day after CFA injection were highly conspicuous compared to those from later periods [21]. These peak responses occurred relatively early when viewed in context of the typical progress of inflammation, where the peak of the acute inflammatory reaction is reached 24–72 h after its initiation, which depends on intercellular communications among immune cell components, tissues and nerves [37].

Pelvic mechanical allodynia quickly developed in mice with urinary tract infections from uropathogenic Escherichia coli (E. coli) strain NU14, but not from asymptomatic bacteriuria strain 83972 [54]. The allodynic effects of the bacterial endotoxin lipopolysaccharides (LPS) collected from these two strains also significantly differed; the transurethral instillation of LPS from NU14, but not that from 83,972, evoked pelvic allodynia that began 1 h post-instillation, suggesting that certain specific chemical components or unique submolecular structure of LPS species may be required for the induction of pain. The development of allodynia occurred earlier than the increase in the levels of neutrophil myeloperoxidase, indicators for inflammation development and the allodynia was blunted in mice lacking in Toll-like receptor type 4 (TLR4), which is an LPS receptor. None of the studies discussed above that examined CFA and uropathogenic E. coli also evaluated the acute neuronal responses to CFA exposure. Very recently, several other pain-producing strains of bacteria were compared with regard to their ability to directly activate sensory neurons, which will be described below [14].

Interest in the effects of pathogenic infection on mood regulation is currently increasing in the field of psychiatry. Intriguing features of murine reactions were collected to gastrointestinal infections with Gram-(−) pathogen Campylobacter jejuni at subclinical levels that may not engage immune activation. Without associated changes in IL-6 level and contents of immune cells such as neutrophils, monocytes, lymphocytes, etc., anxiety-like behaviors such as non-exploratory and grooming behaviors and the preference of closed arm entries on the elevated plus-maze were increased in infected mice [40]. Following studies have proposed that the activation of the vagal sensory afferents may be responsible for these neurological changes and have suggested the bacterial endotoxin LPS to be a candidate for nerve stimulation by demonstrating increased c-Fos-like immunoreactivities, a surrogate measure for neuronal activity in vagal ganglia [23, 24]. In the vagal ganglia and nucleus of the solitary tract, the degrees of c-Fos-like immunoreactivity stayed in the plateau between 5 and 10 h after infection, which again indicates that neuronal activation begins at a time during which the intercellular signaling cascades of the immune cell network have hardly had a chance to mature. Although the outcomes that follow these perceptions are different from what is focused on in this review, early stimulation of sensory neurons by a pathogen could be indicative of the possibility that a similar paradigm works in the somatosensory system. The effects of LPS suggest that sensory neurons may also utilize a pattern recognition mechanism.

#### Neurons also utilize pattern recognition receptors (PRRs)

The innate immune cells express particular types of molecules that bind to a broad spectrum of substances originating from pathogens and injuries such as common exterior patterns of pathogen surfaces, their exotoxins, viral nucleotides and intracellular contents draining from injured tissues. For bacterial pattern recognition, TLRs, nucleotide-binding oligomerization domain (NOD)-like receptors (NLR), and c-type lectin receptors (CLR) constitute the important PRR receptor pool. In particular, TLR1, 2, 4–6 and 9, dectins and mincle receptor, and NOD1 and 2 are key players in recognizing bacteria-specific substances.

The protein encoded by the myeloid differentiation primary response gene 88 (MyD88) commonly mediates the intracellular signal transduction cascade initiated by TLR activation, which results in the transcription of pro-inflammatory cytokines via nuclear factor κB (NFκB) and the MAPK pathway [32]. TLR4 activation can also utilize toll/interleukin receptor -domain-containing adapter-inducing interferon-β (TRIF)-dependent pathway, or extracellular signal-regulated kinase (ERK), p38 MAPK and the c-Jun N-terminal kinase pathways [36]. Activation of NOD1 and 2 subsequently causes the activation of receptor-interacting serine/threonine-protein kinase 2 (RIPK2), which also finally induces NFκB-mediated cytokine transcription. CLRs including dectins frequently use the Src family kinase-spleen tyrosine kinase-NFκB axis but other unknown downstream cascades may be involved. Most intracellular signal transduction is initiated at the cell surface where the receptors are located and is activated by their binding with exogenous ligand substances with the exception of TLR9 (which stays in the endosome) and NLRs (which are located in the cytoplasm).

Sensory neurons may have evolved to express PRRs to directly respond to bacterial pathogens. Under this assumption, the expression of these receptors has been examined in sensory neurons. TLR4 mRNA and immunoreactivity has been detected in the nodose ganglia of rodents, which may contain both neuronal and non-neuronal components [27]. Neuronal TLR4 expression was confirmed in a subset of human and rodent trigeminal ganglionic (TG) sensory neurons that are peptidergic nociceptor neurons expressing the vanilloid subtype 1 TRP ion channel (TRPV1) and CGRP [20, 22, 62]. LPS challenge evoked acute electrical currents from rat trigeminal neurons and sensitized TRPV1 activity after 5 min of treatment [20]. Moreover, 15 min of exposure of rodent TG neurons to LPS facilitated neuronal CGRP release elicited by capsaicin, a TRPV1 agonist, although LPS alone failed to induce peptide release [22]. TLR4 expression was also detected in cultured murine dorsal root ganglionic (DRG) neurons and 24 h-LPS treatment elevated the DRG neuronal expression of nociceptin/orphanin FQ, a centrally acting pro-nociceptive peptide [2]. TLR9 was also shown to be present in human and murine DRG neurons [52]. Sixteen hours of the incubation of DRG neurons in a synthetic oligonucleotide with a CpG motif, which can activate the TLR9 receptor, led to the up-regulation of TLR9 and TRPV1 expression, TRPV1 translocation, Ca^2+^ flux via TRPV1 activation, and the production of prostaglandin E2 (PGE2), chemokine (C-X-C motif) ligand 5 (CXCL5), chemokine (C-X-C motif) ligand 10 (CXCL10), IL-1α and IL-1β. Thermal hypersensitivity, which has been suggested to be mediated by TRPV1 in an animal cancer pain model, was blunted by TLR9 knockdown. In the same study, the expression of TLR3 and TLR 7 was also detected in the sensory neurons. Despite not being neurons, arterial chemosensory glomus cells, which are of neural crest origin and signals to the carotid afferent, a branch of the glossopharyngeal nerve, express TLR2 and 4 [1]. Strong reverse transcriptase-polymerase chain reaction signals were observed for mRNAs of TLR1, 4, 5 and 6 in the colonic DRG neurons of mice, while those for TLR2 and 9 mRNAs were relatively faint [49]. Despite not causing direct excitation, LPS acutely potentiated the excitability of these neurons.

Several studies raised the possibility of NLRs being present in sensory neurons. The mRNAs for NOD1 and NOD2 were detected in mouse colonic DRG neurons, although the signals were relatively moderate compared to those for TLRs [49]. Cryopyrin, which is a NOD-like receptor containing pyrin domain subtype 3 (NLRP3), also known as nacht domain-, leucine-rich repeat- and pyrin domain (PYD)-containing protein 3 (NALP3), is constitutive of inflammasome inside macrophages and appears to detect bacterial RNA [35]. Cryopyrin was recently shown to be expressed in non-peptidergic isolectin B4 (IB4)-bound rat trigeminal neurons [13]. The expression of CLRs in sensory neurons has not been thoroughly studied. Very recently, Schwann cells have been shown to express mannose receptors, which mediate the internalization of Streptococcus pneumonia [41].

Collectively, among the bacteria-detecting PRRs, it is clear that certain types of TLRs exist in nociceptor sensory neurons. However, their role in sensory neuronal excitability is often not immediate and involves an indirect mechanism that appears to depend on the reduction of rheobase than the induction of direct firing. Interestingly, without direct information about which TLR mediates this response, several research groups have investigated whether neurons are directly excited by LPS. Mild (20–50 %) but acute (in 2 min) increases in intracellular Ca^2+^ levels were detected upon exposure to LPS (0.1–10 μg/mL) in cultured rodent DRG neurons [28]. Because these Ca^2+^ increases are dependent on the presence of extracellular Ca^2+^, cation transport by surface Ca^2+^ channels seems to be critical for this reaction. In the same study, 25 min of exposure also evoked CGRP secretion from the sensory neurons, which was mediated by protein kinase A and C. Hou et al. suggested that the influxed Ca^2+^ through the surface transport may activate the enzyme cascade. Diogenes et al. [20] also confirmed that a fast influx of intracellular Ca^2+^ occurs in trigeminal neurons in response to LPS. This Ca^2+^ response partially remained even during TLR4 antagonism. They further demonstrated that LPS elicited a whole cell inward current in cultured trigeminal neuron with a fast component that occurred within a second and a relatively slow component that arose about one minute after the start of LPS exposure [20]. The presence of such a fast and Ca^2+^-involved electrical component implicates the possible existence of an electrogenic receptor system for sensing LPS.

### TRPA1 for LPS

To survey environmental insults, sensory neurons possess unique molecular tools that are often absent in innate immune cells including sensory neuron-specific G-protein coupled receptors and ion channels. TRP ion channel members are in the family of environmental sensor molecules. Mechanical, thermal or chemical challenges stimulate the TRP channels in sensory neurons and their activations rapidly prompt the neuronal firing. In their chemical stimulator pool, lipids are considered important ones. Two major lipid-activated TRP channels are TRPV1 and TRPA1. TRPV1 is bound and activated by lipoxygenase metabolites intracellularly generated during pro-inflammatory signal transduction [18, 29, 51]. TRPA1 covers detection of a more extensive list of lipid species including various lipid peroxidation products, cyclopentenone prostaglandins, nitrative fatty acids and fatty aldehydes containing αβ-unsaturated carbons [16, 66]. In addition, TRPV3 and TRPV4 are also activated by some diphosphoryl isoprenes [5, 6]. The coupling of direct lipid detection and rapid channel activation enables fast pain perception that may help cognition-based avoidance from exposure of harmful lipids. In addition, it may also exacerbate local inflammation since depolarization by TRP activation also causes an antidromic release of pro-inflammatory neuropeptides from the nerve terminals towards the inflamed area (neurogenic inflammation). Given its acute effects on neuronal excitation and lipid-containing structure, LPS can be hypothesized to interact with these TRPs.

Indeed, Meseguer et al. [47] demonstrated that LPS from gram-(−) bacteria activated mouse TRPA1 in trigeminal and nodose ganglionic neurons and mouse and human TRPA1 in heterologous expression systems. Surprisingly, data from TLR4-knockouts and TLR4 antagonism excluded the possibility of the engagement of TLR binding with its downstream signal transduction. Single channel openings in their outside-out patch clamp observation further excluded the possible participations of other cytoplasmic pathways. The negative shift in voltage dependence, accelerated pore opening and decreased deactivation speed of TRPA1 at least partly explain the membrane-delimited mechanisms. TRPA1 is well known to be activated by electrophilic lipids by covalent binding with its N-terminal cytoplasmic cysteine residues. Examination with a cysteine mutant insensitive to the TRPA1 activator allylisothiocynate (AITC) showed that LPS-induced activation is independent of this covalent binding paradigm. Activation occurred in a dose dependence manner where LPS can activate TRPA1 roughly at 0.1 through 100 μg/mL, which is well-matched to the effective concentrations known to elicit acute Ca^2+^ or electrical responses from sensory neurons [20, 28]. Since the molecular weight of LPS is typically between 10 and 20 kDa, TRPA1 activation may require nanomolar to micromolar concentrations of LPS, indicating that the potency of LPS seems to be similar or slightly stronger than those of other known lipidergic activators of TRPA1.

In the same study, Meseguer et al. further showed the structural determinants for the LPS activation of TRPA1. LPS consists of a polysaccharide O-antigen, a core oligosaccharide attached by phosphates and amino acids, and lipid A. The authors predicted that the lipid moiety may determine the agonist activity, as is the case in other lipids that activate TRPA1. Polymyxin B, which contains multiple cationic residues and exerts its antibiotic action by neutralizing anionic lipid A, interfered with the activation of TRPA1 by LPS, whereas minimal effects were observed on AITC action. Moreover, purified or synthetic lipid A retained the capacity to activate TRPA1. Interestingly, dependent on the structure of lipid A, LPS displayed different TRPA1-activating potentials. LPS molecules with asymmetrical hexa-acyl lipid A, which typically constitutes the cell walls of E. coli, Salmonella typhimurium and Klebsiella pneumonia, displayed a stronger potency than those with asymmetrical penta-acyl lipid A (e.g. those found in Serratia marcescens and Pseudomonas aeruginosa), or with symmetrical hexa-acyl lipid A (e.g. those found in Neisseria meningitides and Salmonella Minnesota). Despite being a competitive antagonist for TLR4, LPS from Rhodobacter sphaeroides could activate TRPA1. These results are reminiscent of the strain-dependent occurrence of pelvic allodynia in urinary tract infections of E. coli mentioned above [54]. Even within the same bacterial species, structural difference between lipid A moieties possibly cause significant difference in terms of pain production when acutely instilled.

The TRPA1-dependent activity of LPS on the excitation of sensory neurons has also been confirmed in the in vivo examinations for generation of pain and neurogenic inflammation. Surprisingly, when TLR4 knockouts were assessed, it was found that TLR4 contribution was ignorable to the mechanical allodynia that occurred within 24 h post LPS injection as compared to the contribution of TRPA1. Therefore, it was suggested that the nociceptor-specific TRP channels detect LPS as soon as some of Gram-(−)-pathogens invade, and the activation of TRP channels leads to acute pain sensation and an early phase of inflammation. It is still possible that there is a TLR-mediated mechanism but that the TLR effect may be redundant since its signaling pathway may merge into a TRPA1-dependent one or not sufficient to induce an acute reaction. Such nociception occurs as fast as minutes to several hours after LPS is measurable in tissues, suggesting that hosts earn a critical time period for their protection, which is uniquely earlier than ~24 h, the time when the inflammatory type protection in which the innate immune system take the lead becomes active.

### ADAM10 for α-hemolysin

**Fig. 1:**
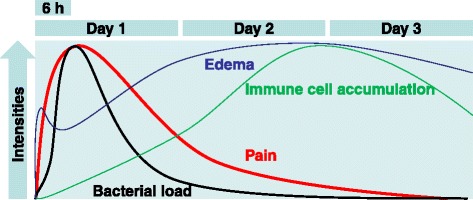
Summary of the time course of tissue responses to S. aureus infection observed by Chiu et al. [14]. The progress of pain intensities better seem to correlate that of bacterial load than those of other parameters. This figure is modified from Supplemental figure 4 of Chiu et al. [14]

Chiu et al. established solid basis for the field of the pathogen-mediated pain by completing pain profiles from 0 to 72 h after infection [14]. They observed for the first time that the mechanical and thermal pain hypersensitivities of the experimental animals rapidly developed within 6 h after infection with Staphylococcus aureus (S. aureus), and was independent of the immune response profiles that dominated from 24 to 72 h after infection [Fig. 1]. This result supports previous less comprehensive data on the neural responses to infection at early time points mentioned above [21, 23, 24, 54]. TLR-dependent mechanism for this rapid response was again excluded in their hands using MyD88- or TLR2-deficient mice. Cultured nociceptive C-fiber neurons acutely exhibited elevations of intracellular Ca^2+^ and membrane potentials in response to bacterial treatments. On this basis and after further screening for nociceptive elements, two unique mechanisms for acute sensory neuronal excitation by pathogens were proposed. The first involves the interaction between an exotoxin and a disintegrin and metalloproteinase domain-containing protein 10 (ADAM10).

Some exotoxins are peptides that are able to form pores, such as cytolysins and hemolysins. When incorporated in the surface membrane of host cells, they may perturb the intracellular signal transduction of the host cells by abnormally transporting particles or ions, or by inducing cell lysis, which promotes pathogen spread and inhibits leucocyte function [19, 61]. In the late 1990s, the three-dimensional structure of pore-forming toxins began to be elucidated, along with the mechanism by which ions are conducted through the pore in the plasma membrane of live cells [46, 57]. However, it has still remained unclear whether these pore-forming exotoxins can be inserted into neurons, form pores and cause neuronal excitation by increasing ionic conductance across these pores. Eventually, Chiu et al. demonstrated the electrogenic effect of α-hemolysin. Cultured DRG neurons fired action potentials and displayed increased intracellular Ca^2+^ levels within 2–10 min after exposure to α-hemolysin in hundreds of nanomolar levels. The intraplantar injection of α-hemolysin at nanograms produced pain within a similar time period and furthermore, caused mechanical, heat and cold hypersensitivity, which peaked at 6–7 h post-injection.

Upon exposure to micromolar or millimolar quantities of toxins the host cell membrane may nonspecifically and passively absorb those, but the potency or affinity of such nanoscale levels of toxin is known to require specific binding to host receptors [10, 11, 25]). The membrane-bound sheddase ADAM10 plays diverse roles in the central nervous system including amyloid production and cadherin cleavage, but little is known about its role regarding the sensory function. Recently, ADAM10 was shown to play the role of a receptor and even promoted the oligomerization and localization of the α-toxin ([63]; Inoshima et al., 2011). ADAM10 has consistently been shown to be critical for the neuronal excitatory effects of α-toxin. The pore assembly of α-hemolysin depends on the presence of ADAM10, whose expression was confirmed by Chiu et al. in a subset of Nav1.8-Cre/TdTomato-positive nociceptors of the DRG sensory ganglia. The H35L mutant of α-hemolysin, which cannot form the heptameric pore, failed to elevate intracellular Ca^2+^, evoke action potentials in DRG neurons, and induce acute pain. This suggests that the pore forming ability and electrogenecity of α-hemolysin may be important for nociceptor excitation. For different hemolysin species, besides the pore-mediated conductance, other mechanism of actions including mobilization of Ca^2+^ transporting components are also conceivable as an alternative pathway. Activation of the store-operated mechanism mediated by stromal interaction molecule (STIM) and the calcium release-activated calcium modulator (Orai) system via a unknown signal transduction has been suggested to contribute to increases in neuronal Ca^2+^ by Jover et al. [34].

### Formyl peptide receptor 1 for bacterial formyl peptides

Other virulence factors may interact with and activate sensory neurons as well as pore-forming toxins. Chiu et al. showed that α-hemolysin-deficient S. aureus mutants still cause moderate hyperalgesia. Proteinaceous and oligomeric toxins may be chemically vulnerable to heat. The application of heat-treated bacteria resulted in neuronal excitation and nocifensive behaviors. Thus, noxious substances from bacteria may be among heat-stable factors. In the list for positive hits in Chiu’s screening of relatively heat-stable components, peptidoglycans and lipoteichoic acid which are TLR ligands were eliminated, again indicating that TLR engagement is less possible. On the other hand, some bacterial ligands for G-protein-coupled receptors (GPR) were elected: a series of N-formyl peptides. The presence of prokaryote-specific components that are not found in host eukaryotes can be hypothesized to be useful parameters for the host to identify pathogen invasion. Exotoxins including hemolysins and cell wall components including LPS are the best examples. Formyl peptides are also among those. When bacteria synthesize proteins, they start with formyl methionine, which is only used for organellar synthesis in eukaryotes. Innate immune cells detect these N-formylated peptides using the formyl peptide receptors (FPRs) which are GPRs, and exert a chemotactic event to clear the sources that generate the peptides [8]. The optimal peptide length for maximal FPR activation appears to be 2–6. As previously mentioned, formyl peptides are also synthesized in host organelles that have prokaryotic origins including mitochondria. These host cell-derived formyl peptides can be accidentally secreted when cells are damaged. Thus these formyl peptides drained from host cells are considered as damage signals as ATP, other nucleotides and potassium ions are. Innate immune cells can contribute their protective inflammatory processes to the neutralization of the damaged situation in a similar manner observed in infection [15]. Recent research has showed that FPRs can detect other peptidergic or lipidergic messengers that are important for the intercellular communication between immune cells [17].

The presence of FPRs and the rapid responses of DRG sensory neurons by pathogenic formyl peptides were first demonstrated by Chiu et al. [14]. Bacterial formylated peptides such as fMLF from E. coli and fMIFL from S. aureus induced increases in intracellular Ca^2+^ in capsaicin and AITC-responsive nociceptor sensory neurons (which were putatively TRPV1- and TRPA1-positive) at 1 μM as compared to the negligible response to the unformylated controls. Typical GPR downstream signaling related to phopholipase C or A2-mediated intracellular Ca^2+^ mobilization or cation influx may explain those neuronal responses [8, 9, 12]. Nocifensive behaviors also developed in the mice intraplantarly injected with formyl peptides. Two interesting features were that the peak phase was achieved relatively quickly (between 0.5 and 3 h) as compared to what was observed after the administration of whole bacteria or exotoxin (~6 h), and that only the mechanical phenotype was affected. Since TRPA1-positive neurons were responsible for formyl peptide responses and TRPA1 has been shown to directly mediate noxious mechanosensation, FPR-TRPA1 coupling for the rapid sensory excitation by formyl peptides might be conceivable but remains to be clarified. In vitro responses were abrogated by FPR1 antagonist and in vivo responses were blunted in FPR1-deficient mice, both of which indicate the importance of the FPR1 receptor. Strong FPR1 mRNA signals were confirmed in Nav1.8-Cre/Tomato-positive nociceptor neurons. Based on the recent findings by Chiu et al., ADAM10 and FPR1-mediated neuronal detections may cover more extensive chances of infection than those detected by the interactions between LPS and TRPA1 since pore-forming toxins and formyl peptides are generated by both Gram-(−) and (+) bacteria.

### Effects of direct pathogen detection on immune activities

As a next step, it would be wondering how these rapid neuronal sensations affect the immune system. It has been shown that outcomes from the sensory neuronal detection of a noxious environment have two important features at the viewpoint of the host defense: the local promotion of neurogenic inflammation and the systemic accommodation of immune responses. The terminal arborization of peptidergic nociceptor neurons exerts an axon reflex upon excitation by generating neuropeptides such as substance P and CGRP onto the injured tissues. These peptides have pro-inflammatory roles, directly promoting immune cell mobilization and function and indirectly facilitating access into sites of injury via vasodilation and increases in vascular permeability. The stimulation of neurons by cytokines and pro-inflammatory mediators released from immune cells (which have traditionally been considered as the prime candidates for rapid pain producing mechanisms before the discovery of the TRP/ADAM/FPR-mediated mechanisms) amplifies these interactions, establishing a vicious cycle of neurogenic inflammation [15, 30]. On the other hand, a typical reflex arc through the CNS may negatively control inflammation at a systemic level. Similar to sensory-motor reflexes involved in motions, heart beats and digestion, excessive sensory input that can threaten body homeostasis appears to automatically cause an adjustment that leads to a restoration. The nematode version and mammalian vagal versions of this reflex onto the immune system against excessive immune activation have been reported [4, 53, 59, 64].

Evidence for the somatosensory version of this reflex was provided by Chiu et al. and was determined by investigating animals in which the sensory arc is removed. Nav1.8-Cre/diphtheria toxin A (DTA) transgenesis results in toxin-mediated ablation of the Nav1.8-lineage nociceptors which are the majority that relays pathogenic pain signals via the receptors introduced above. As expected, the inoculation of bacteria did not affect the pain states of these transgenic animals. Twenty-four hours after injection, the popliteal lymph nodes that drain the injected plantar pad were enlarged, both in weight and cell counts (mainly B and T cells and monocytes) as compared to the wild type littermates, although spleen size was not affected. The tissue levels of TNF-α which dominates the node hypertrophy were also more increased. Therefore, the loss of sensory information may result in the reflex arc becoming non-functional, leading to an out-of-control situation. The specific participants of the CNS and output arcs that tune this difference need to be explored in the future. It is hypothesizable that tissue inflammatory indices may be maintained since the loss of local neurogenic powers due to nociceptor ablation can be complemented by the nodal hyperactivation above, and in fact, slightly more increased tissue swelling, significantly greater monocyte infiltration and comparable neutrophil infiltration were observed in Nav1.8-Cre/DTA mice.

With regards to local neurogenic inflammation, the outcomes from experiments with neuropeptide treatment contradict the myth of its pro-inflammatory action. Expectedly, S. aureus supernatant and α-hemolysin induced CGRP release from cultured DRG neurons. However, cultured peritoneal macrophages secreted significantly smaller amounts of TNF-α on exposure to heat-killed bacteria when incubated with CGRP, galanin or somatostatin, all of which were top ranked peptides in their microarray analyses both in terms of peptide levels in nociceptors and receptor levels in innate immune cells. This suppressive effect of CGRP was confirmed in bone marrow macrophages after Gram-(+) endotoxin lipoteichoic acid stimulation. Moreover, CGRP injection did not alter the inflammation at local sites of infection during S. aureus infection. Instead, CGRP treatment reduced the cell count in the draining lymph nodes. Consequently, the collection of data from Chiu et al. on local and systemic neural reflexes indicates that these two mechanisms consistently down-regulate host defense. In fact, the majority of studies have demonstrated the positive effects of neuropeptides on immune activation (for review: [15]; McMahon et al., 2015), but a number of recent reports on inhibitory modulation have also been increasing (for review: [26]). As opposed to vascular effects which have been shown to be relatively consistent, immune modulations seem to require more sophisticated investigative approaches tailored to individual cell types, inflammatory phases and signaling cascades.

### Extended questions

Despite the recent important findings summarized above [Table 1], the studies dealing with direct pathogen-nerve interactions are still at their early stages. Many related questions and other possibilities remain for unveiled receptor interactions that may further account for pain-mediated protection or adaptive mechanisms.

**Table 1:** Summary of Atypical sensors and their ligands originating from bacterial pathogens

Sensors	Painful substances	Source bacteria	Gram-staining categories	References
FPR1	N-formyl peptides fMIFL	Staphylococcus aureus	(+)	Chiu et al. [14]
FPR1	N-formyl peptides fMLF	Streptococcus pneumoniae	(+)	Chiu et al. [14]
FPR1	N-formyl peptides fMIVIL	Listeria monocytogenes (predicted)	(+)	Chiu et al. [14]
FPR1	N-formyl peptides fMLF	Escherichia coli	(−)	Chiu et al. [14]
TRPA1	LPS (lipid A)	Escherichia coli	(−)	Meseguer et al. [47]
TRPA1	LPS (lipid A)	Salmonella typhimurium	(−)	Meseguer et al. [47]
TRPA1	LPS (lipid A)	Klebsiella pneumonia	(−)	Meseguer et al. [47]
TRPA1	LPS (lipid A)	Serratia marcescens	(−)	Meseguer et al. [47]
TRPA1	LPS (lipid A)	Pseudomonas aeruginosa	(−)	Meseguer et al. [47]
TRPA1	LPS (lipid A)	Rhodobacter sphaeroides	(−)	Meseguer et al. [47]
ADAM10	α-hemolysin	Staphylococcus aureus	(+)	Chiu et al. [14]
AT2	mycolactones	Mycobacterium ulcerans	(+)	Marion et al. [42]

#### Can the role of the TLR be excluded?

It is surprising that the contribution of the TLR-MyD88 system to pathogenic pain induction was negligible given its primary role in providing surveillance against bacterial invasion and its well-documented presence in sensory neurons. Although TLR machinery scarcely depolarizes neurons directly but rather likely tends to sensitize them, the sensitization itself might still be important, when we consider that EP/DP receptors for prostaglandins (for TRPV1 activity) and interleukin-1 receptors (for voltage-gated Na^+^ channel activity) operate in the sensory neurons in a sensitization-dependent manner, contributing to profound and rapid exacerbation of pain [7, 48]. Moreover, the six hours it takes for the pain sensation to reach its peak appears to be enough time for the sensitization mechanism to development of pain [20, 22]. One possible explanation for the underwhelming effects of TLR activation is that the downstream signal transduction may be redundant given the action of the atypical receptors. The effect of TLR effect may be masked if the signal is merged with the transduction of the same effectors, for example, TRPA1 or other surface cation channels. Interestingly, the activation of TLR7, which may utilize similar signaling cascades as other TLRs but is known to be less important in bacterial infection and located in organelles, led to the immediate excitation of sensory neurons [39, 50]. The final effector for neuronal excitation by the TLR7 pathway seems to be TRPA1. S. aureus and E. coli are major causes of painful infectious diseases, which were mainly tested in the two important studies conducted by Chiu et al., and Meseguer et al. It would be interesting to study whether TLRs have greater contribution to the pain response to other pathogenic species with different pain-producing virulence factors.

### Other unknown interactions

It remains to be determined which NLRs and CLRs are abundantly expressed in nociceptor neurons and to what degree the subtypes already known to be present in those neurons including NOD1, NOD2 and cryopyrin, contribute to the exacerbation of pain or neuroinflammation. Similar to the case of TRPA1, the resources for ‘innate’ neurosensory receptor pool might be utilized for pathogen detection. Recently, the bitter taste receptor TAS2R38, expressed in the upper respiratory epithelium, was shown to be activated by Gram-(−) quorum-sensing molecules including N-butyryl-L-homoserine lactone and N-3-oxo-dodecanoyl-L-homoserine lactone in micromolar concentrations [38]. This activation causes Ca^2+^ influx into cells, nitric oxide production and the propulsion of the motile cilia, finally resulting in the increased clearance of pathogens and suggesting that the outcomes of this mechanism are not limited to a detection but include a protective reflex without an intercellular circuit. It would be interesting to assess whether somatosensory or vagal nociceptors share the expression of these bitter taste receptors. In addition, the report by Lee et al. suggests that the virulence factors detectable by the host may become enlarged to ones that are utilized for interbacterial messengers.

#### For whom is the painful interaction with neurons more profitable, host or pathogens?

In terms of pathogen-induced direct pain, the degree of pain may differ between pathogen species: for example, the acute pain parameters after inoculation with heat-killed Mycoplasma fermentans or E. coli, were lower than those after inoculation with S. aureus and S. pneumoniae [14]. In addition, some infectious diseases such as impetigo, cutaneous anthrax, syphilis, scrofuloderma and Buruli ulcer (also known as Bairnsdale ulcer and Daintree ulcer) are often not accompanied by pain in the early phases of infection despite the severity of tissue damages. In particular, mycobacterium ulcerans, which causes the Buruli ulcer, appears to actively subvert the acute pain response by secreting macrolide exotoxins known as mycolactones. Nanomolar and micromolar concentrations of mycolactones have been shown to activate sensory neuronal angiotensin type II receptor (AT2), which employs the Gi coupled-phopholipase A2 pathway. This activation causes the opening of two-pore-domain background K^+^ channels, eventually hyperpolarizing nociceptor sensory neurons [42]. Since these exotoxins also suppress the immune reaction, this pathogen seems to take a strategy to avoid both of the two important axes of the host vigilance for their soft landing. On the other hand, because the stimulation of sensory nociceptors during the early phase contributes to both local and systemic immune evasion, as shown in the case for S. aureus presented by Chiu et al., the production of acute pain can also ultimately help pathogen invasion. Further observations are needed to understand what such seemingly opposed strategies that have been evolutionarily selected for pathogen survival. It is possible that the studies regarding this issue may discover other unknown atypical sensors and analgesic targets as the angiotensin type II receptor was the case.

## Conclusions

Recent efforts to observe the rapid interactions between bacterial pathogens and sensory neurons have discovered several atypical and direct contact points: ADAM10 for α-hemolysin, FPR1 for bacterial peptides, TRPA1 for LPS, etc. These findings have led to two possible hypotheses: sensory neurons may promote cognitive detection and preparation of the host for eliminating the pathogenic intruders, or pathogens may utilize these interactions with sensory neurons to down-regulate the innate immune defense of the host by stimulating systemic and local feedback circuits. Future observations are needed to extend other neuronal mechanisms interacting with each of diverse and particular phyla in the bacterial domain and even with other pathogenic microbes such as fungi and viruses. Because such interactions may proceed under tight communication with the immune system, triangular perspectives that all cover the three parties of pathogens, nerves and immune cells will enrich our symbiotic insights and contribute to devising of protective strategies.

## Ethics approval

This study does not need an approval of an ethical committee or consent for publication.

## Abbreviations

ADAM: disintegrin and metalloproteinase domain-containing protein
AITC: allylisothiocynate
AT2: angiotensin type II receptor
CFA: complete Freund’s adjuvant
CGRP: calcitonin gene-related peptide
CLR: c-type lectin receptor
CXCL: chemokine C-X-C motif ligand
DRG: dorsal root ganglion
DTA: diphtheria toxin A
E. coli: Escherichia coli
ERK: extracellular signal-regulated kinase
FPR: formyl peptide receptor
GPR: G-protein-coupled receptor
IB4: isolectin B4
IL-1β: interleukin-1β
LPS: lipopolysaccharide
MAPK: mitogen-activated protein kinase
MyD88: myeloid differentiation primary response gene 88
NALP: nacht domain-, leucine-rich repeat- and pyrin domain-containing protein
NFκB: nuclear factor κB
NGF: nerve growth factor
NLR: nucleotide-binding oligomerization domain-like receptor
NLRP: nucleotide-binding oligomerization domain-like receptor containing pyrin domain
NOD: nucleotide-binding oligomerization domain
Orai: calcium release-activated calcium modulator
PG: prostaglandin
PI3K: phosphoinositide 3-kinase
PRRs: pattern recognition receptors
PYD: pyrin domain
RIPK: receptor-interacting serine/threonine-protein kinase
S. aureus: Staphylococcus aureus
STIM: stromal interaction molecule
TG: trigeminal ganglion
TLR: toll-like receptor
TNF-α: tumor necrosis factor-α
TRIF: toll/interleukin receptor -domain-containing adapter-inducing interferon-β
TRP: transient receptor potential
